# How To Perform Meaningful Estimates of Genetic Effects

**DOI:** 10.1371/journal.pgen.1000062

**Published:** 2008-05-02

**Authors:** José M. Álvarez-Castro, Arnaud Le Rouzic, Örjan Carlborg

**Affiliations:** Linnaeus Centre for Bioinformatics, Uppsala University, Uppsala, Sweden; University of Alabama at Birmingham, United States of America

## Abstract

Although the genotype-phenotype map plays a central role both in Quantitative and Evolutionary Genetics, the formalization of a completely general and satisfactory model of genetic effects, particularly accounting for epistasis, remains a theoretical challenge. Here, we use a two-locus genetic system in simulated populations with epistasis to show the convenience of using a recently developed model, NOIA, to perform estimates of genetic effects and the decomposition of the genetic variance that are orthogonal even under deviations from the Hardy-Weinberg proportions. We develop the theory for how to use this model in interval mapping of quantitative trait loci using Halley-Knott regressions, and we analyze a real data set to illustrate the advantage of using this approach in practice. In this example, we show that departures from the Hardy-Weinberg proportions that are expected by sampling alone substantially alter the orthogonal estimates of genetic effects when other statistical models, like F_2_ or G2A, are used instead of NOIA. Finally, for the first time from real data, we provide estimates of functional genetic effects as sets of effects of natural allele substitutions in a particular genotype, which enriches the debate on the interpretation of genetic effects as implemented both in functional and in statistical models. We also discuss further implementations leading to a completely general genotype-phenotype map.

## Author Summary

The rediscovery of Mendel's laws of inheritance of genetic factors gave rise to the research field of Genetics at the very beginning of the last century. The idea of traits being determined by the effects of inherited genes is thus the conceptual core of Genetics. After more than one century, however, we still lack a completely general mathematical description of how genes can control traits. Such descriptions are called genotype-phenotype maps, or models of genetic effects, and they become particularly cumbersome in the presence of interaction among genes, also referred to as epistasis. The models of genetic effects are necessary for unraveling the genetic architecture of traits—finding the genes underlying them and obtaining estimates of their individual effects and interactions—and for meaningfully using that information to investigate their evolution and to improve response to selection in traits of economical importance. Here, we illustrate the convenience of using a recently developed model of genetic effects with arbitrary epistasis, NOIA, to inspect the genetic architecture of traits. We implement NOIA for practical use with a regression method and exemplify that theory with a real dataset. Further, we discuss the state of the art of genetic modeling and the future perspectives of this subject.

## Introduction

There is an increasing interest in Quantitative Genetics and Evolutionary Biology to identify genetic effects, and more particularly gene interactions, on a genome-wide scale and to understand its role in the genetic architecture of complex traits [1],[2]. Genome scans for quantitative trait loci (QTL) have proven to be a successful strategy for identifying genetic effects and interactions. Two of the main issues in the development of QTL mapping methods are which models of genetic effects to use and how to test for effects in regions between marker locations. The second issue is important not only for considering the genome as a virtually continuous space where to map the QTL, but also to efficiently analyze incomplete data sets, which are the norm in practice [3]. Lander and Botstein [4] developed the classic interval mapping (IM) method, in which they showed how to perform a QTL mapping strategy implemented with the most likely genotypes for the genome regions in between marker locations, given the genotypes at the flanking markers. This method has been extended in several ways [5]–[8]. Albeit the computation of those likelihoods is complex and time demanding, Haley and Knott [9], (see also [10]) provided a convenient approximation of them by means of a simple regression method.

Regarding now the first issue mentioned above—the models of genetic effects—the definition of the genetic effects in Haley and Knott's [9] regression (hereafter HKR) comes from a model that has been extensively used in Quantitative Genetics, the F_∞_ model [11],[12]. However, other models of genetic effects have recently been shown to be more appropriate in QTL mapping. The genetic effects depend not only on the genotypic values but also on the genotype frequencies of the analyzed population (e.g. [13]–[16]). By taking into account these frequencies, it is possible to build orthogonal models that are convenient for several reasons [13]–[19]. First, orthogonal estimates do not change in reduced models, which considerably facilitates model selection for finding the genetic architecture of traits. Second, the estimates of genetic effects obtained by orthogonal models are meaningful in the population under study—they provide the effects of allele substitutions in that population. Third, they directly lead to a proper, orthogonal decomposition of the genetic variance from which to compute important measures, like the heritability of that trait in that population. The statistical properties of HKR could therefore be improved by implementing it with a genetic model that is orthogonal for any possible genotype frequencies in the population under study.

The statistical formulation of the recently developed NOIA (Natural and Orthogonal InterActions) model of genetic effects is orthogonal in situations where previous models are not—for departures from the Hardy-Weinberg proportions (HWP) at any number of loci—and it is therefore more appropriate choice for estimating genetic effects from data in genetic mapping [16]. Furthermore, a novel feature of NOIA is its implementation to transform the genetic effects estimated in the population under study, in two ways. First, they can be transformed into how they would look like in a population with different genotype frequencies at each locus, like an ideal F_2_ population or into an outbred population of interest. Second, using the functional formulation of NOIA, it is possible also to express the genetic effects as effects of allele substitutions from reference individual genotypes—instead of from population means like in the statistical formulation. In other words, starting from the orthogonal genetic effects of a population or sample under study, which are the ideal ones for performing model selection and have a particular meaning, NOIA enables us to obtain the values of the genetic effects that are associated to other desired meanings and are useful, therefore, to inspect different aspects of the evolution of a population, or selective breeding for increasing or decreasing a trait values.

Our motivation for this communication is to show how to use models of genetic effects to obtain estimates of genetic effects from data that have the desired meaning of any particular scientific purpose. To this end we first inspect how much of a difference it makes to use the classical models for ideal populations, such as ideal F_2_ populations, to compute genetic effects in a non-ideal situation, under departures from the HWP. We address this issue by generating simulated populations that depart from the HWP in several degrees and analyzing them with NOIA and other models. We quantify the deviances from orthogonal estimates due to using models that assume ideal conditions in the populations under study, thus showing the practical convenience of using the NOIA model for performing real estimates of genetic effects in QTL experiments. Second, we develop an implementation of NOIA with HKR, allowing it for immediate practical use and illustrate its performance using an example with real data. By this example we provide estimates of genetic effects with different meanings and, for the first time, functional estimates of genetic effects—using an individual genotype as reference—from a real data set. We discuss on how this feature opens new possibilities of using real data to analyze important topics in Evolutionary Genetics.

## Results

### Genetic Models under Departures from Hardy-Weinberg

**Figure 1:**
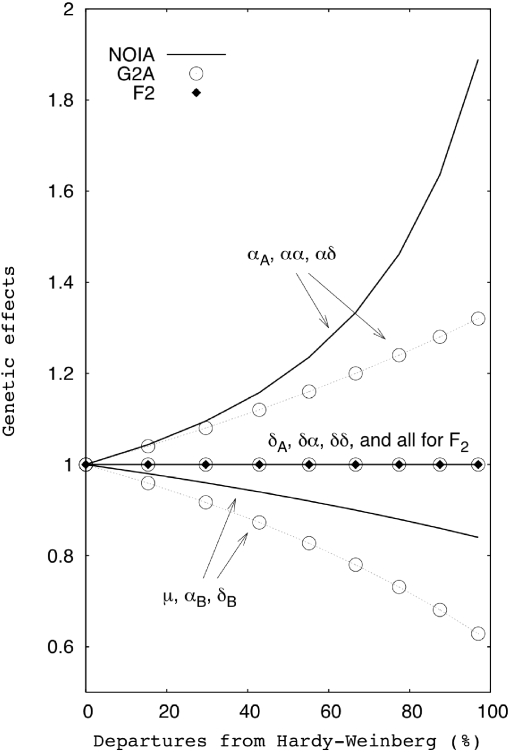
Effects of departures from the HWP on genetic effects. The genetic effects were obtained using the F_2_, G2A and NOIA models in a two locus genetic system that was simulated in nine F_2_ populations with departures from HWP ranging from zero to 97% (see text for details).

Figure 1 shows the results of estimating, with three different models (NOIA, G2A and F_2_), the genetic effects of a two-locus and two-allele genetic system (Table 1) in nine simulated populations under linkage equilibrium (LE) with various degrees of departure from the HWP (see Methods). The eight genetic effects plus the population mean in the only model that is orthogonal in all simulated populations—the statistical formulation of the NOIA model—respond to the increasing departures from HWP in three groups. The first and most influenced group contains the three genetic effects involving the additive effect of the locus affected by departures from HWP, α*_A_*, αα, and αδ. These genetic effects increase substantially with increasing departures from HWP and are doubled when the homozygote *A*_2_ *A*_2_ is almost completely absent. The second group contains the reference point—the mean of the population, μ—and the single locus effects of locus *B* (the one at HWP), α*_B_* and δ*_B_*. The estimates in this group decreased with increasing departures from HWP. The third group contains the remaining three genetic effects, δ*_A_*, δ*α* and δδ, whose estimates are not affected by departures from HWP at locus *B*. The genetic effects measured by the G2A model show the same qualitative behavior described above for NOIA (i.e. also responds in three distinct groups), but are quantitatively different. The reason for this is that G2A can adapt the measurements to the changes in the allele frequencies of the population, but not to the precise departures of the genotype frequencies from the HWP. The genetic estimates obtained using the F_2_ model always give the same values independently of the genetic constitution of the population. The F_2_ thus fails to capture the effects of departures from HWP at all. Thus, unless when the studied population is an ideal F_2_ (and the deviances from HWP are zero, see Figure 1), the estimate of the population mean from G2A and F_2_ is biased and the genetic estimates do not reflect the average effects of allele substitutions in the population under study. Those deviations become more severe as the departure from HWP increases (Figure 1).

**Table 1:** Genotype-phenotype map of the two-locus system used in the simulated populations to evaluate the effect of departures from HWP on genetic effects estimated using the F_2_, G2A and NOIA models.

	Genotype at locus *B*		
Genotype at locus *A*	*B*_1_ *B*_1_	*B*_1_ *B*_2_	*B*_2_ *B*_2_
*A*_1_ *A*_1_	0.25	−0.75	−0.75
*A*_1_ *A*_2_	−0.75	2.25	2.25
*A*_2_ *A*_2_	−0.75	2.25	2.25

**Figure 2:**
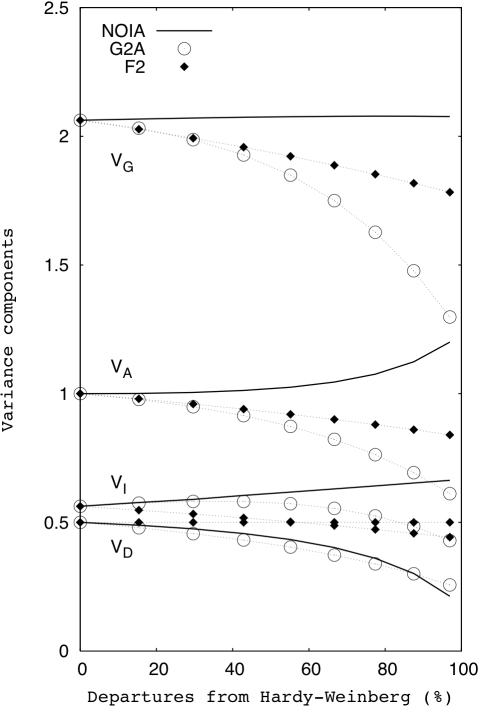
Effects of departures from the HWP on the variance components. The variance decomposition was performed for the same cases as in Figure 1. V_P_ is the phenotypic variance, which (in absence of environmental variance) is equal to V_G_, the genetic variance. V_A_ is the additive variance, V_D_ is the dominance variance and V_I_ is the epistatic (interaction) variance.

Figure 2 shows the variance component estimates obtained in the nine simulated populations, which were obtained by computing the variance over the individuals of the sample population of the correspondent genetic effects (additive effect at locus *A*, additive effect at locus *B*, etc). For orthogonal models, the sum of the three components of variance gives the total genetic variance—which in this case equals the phenotypic variance, since there is no environmental variance in the simulated populations. Here, this is only observed for the variances computed using NOIA. The other two models are not orthogonal in the populations under study (except in the ideal F_2_ population, where the three models coincide), and thus there exist covariances between the genetic effects that would need to be accounted for to obtain the true genetic variance of the population [20]. The decomposition of the genetic variance made by the G2A and F_2_ models is, thus, non-orthogonal. The G2A leads to a greater departure form an orthogonal decomposition of variance than the F_2_ model by the particular kind of departures from HWP simulated here. Both the G2A and F_2_ models underestimate the additive variance and therefore also the heritability of the trait in the simulated populations.

### An Example Using Experimental Data

For illustrating the advantage of using NOIA for analyzing experimental data, we reanalyze a two-locus (*A* and *B*) genetic system with epistasis affecting growth rate in an F_2_ cross between Red junglefowl and White leghorn layer chickens [21]. The two loci are on different chromosomes, thus avoiding linkage disequilibrium (LD). Locus *A* departs significantly from the HWP when considered alone, but not when correcting for multiple testing (see Methods). Table 2 shows the genetic effects and the components of variance for this two-locus system using several models of genetic effects—NOIA, G2A, F_2_ and F_∞_. As explained in the previous subsection, NOIA is orthogonal under departures from the HWP, whereas the other models are not. The F_∞_ model deviates severely from the estimates obtained by NOIA. Deviations are expected since the F_∞_ model is non-orthogonal even in an ideal F_2_ population with no deviations from the expected frequencies due to sampling errors. The F_2_ and G2A models, on the other hand, would be orthogonal under ideal circumstances and the observed deviations from orthogonality of those models when analyzing these experimental data are due to sampling (as explained above). Table 2 shows that the estimates obtained using F_2_ and G2A differ substantially from these of NOIA (up to 18/42% for the G2A and 53/138% for the F_2_ model, for the genetic effects/variance component estimates). This example with real data, thus, shows that it makes a substantial improvement to use NOIA to compute genetic effects and variance decomposition in QTL mapping experiments over the classical models of genetic effects designed to fit ideal experimental situations.

**Table 2:** Estimates of statistical genetic effects (to the left of each cell) and components of the genetic variance (to the right) for an epistatic QTL for growth rate pair in a Red junglefowl×White leghorn layer intercross [21] using four different models.

	Vector of genetic effects, E, and components of variance associated to each of the genetic effects								
Model	μ^1^	α*_A_*	δ*_A_*	α*_B_*	δ*_B_*	αα	αδ	δα	δδ
NOIA	269.49 | 169	1.00 | 0.45	6.74 | 11.28	4.47 | 9.75	−11.75 | 34.32	9.67 | 20.78	−20.30 | 46.66	8.22 | 8.18	−24.80 | 37.87
G2A	269.32 | 164	1.18 | 0.64	7.00 | 12.25	4.15 | 8.43	−10.74 | 28.66	9.68 | 20.83	−20.21 | 46.28	8.28 | 8.35	−24.80 | 38.19
F_2_	269.68 | 177	1.53 | 1.07	7.44 | 13.84	4.90 | 11.80	−11.15 | 31.08	10.48 | 24.76	−19.70 | 44.56	9.50 | 11.07	−24.80 | 38.44
F_∞_	265.23 | 581	11.38 | 59.46	19.84 | 212.83	0.15 | 0.01	1.25 | 0.80	10.48 | 24.76	−19.70 | 90.72	9.50 | 23.94	−24.80 | 169.37

1 The variances in this column are the total genetic variances computed as the sum of the components of variance given in the rest of the columns.

### Transformation To Get Functional Genetic Effects

From the statistical estimates in Table 2, we have computed functional estimates of genetic effects using an analogous expression to (S6), shown in Text S1, derived by Álvarez-Castro and Carlborg [16]. The variances of the statistical estimates can also be transformed to give the variances of the functional estimates using (6), as derived in the Methods section. Choosing “*A*_1_ *A*_1_ *B*_1_ *B*_1_” as reference genotype, the estimates of functional genetic effects, and the standard deviations associated to these estimates, are shown in Table 3. Whereas statistical genetic effects describe the average effects of allele substitutions in a population, functional genetic effects describe the genotype-phenotype map as a series of allele substitutions performed in the genotype of a particular—reference—individual genotype [16],[22], in this case the genotype of the Red junglefowl, “*A*_1_ *A*_1_ *B*_1_ *B*_1_”.

**Table 3:** Estimates of functional genetic effects from the reference of genotype *A*_1_ *A*_1_ *B*_1_ *B*_1_, *G*_1111_±σ*_G_*_1111_ = 265.18±8.35 grams, and their standard deviations for an epistatic QTL pair for growth rate in a Red junglefowl×White leghorn intercross [21].

	*B* 2	
*A* 1	*a_B_ = −10.33*	*d_B_* = 20.95
	σ*_aB_* = 6.24	σ*_dB_* = 10.63
*a_A_* = 0.90	*aa* = 10.48	*ad* = −19.70
σ*_aA_* = 5.96	σ*_aa_* = 4.71	σ*_ad_* = 7.75
*d_A_* = 10.34	*da* = 9.50	*dd* = −24.80
σ*_dA_* = 9.01	σ*_da_* = 6.76	σ*_dd_* = 11.27

1 QTL on chromosome 2 (486 cM).

2 QTL on chromosome 3 (117 cM).

To illustrate the usefulness of these functional genetic effects for understanding how epistatic effects can contribute to phenotype change, we consider the role of this QTL pair in increasing the growth rate in the Red junglefowl. For simplicity, we assume hereafter that *A* and *B* are the only two loci affecting growth rate. From the marginal genetic effects in Table 3, it can be deduced that the White leghorn layer allele at locus *A* slightly increases the phenotype whereas the White leghorn allele at locus *B* actually decreases it, when considered in homozygotes. However, the dominance effects are positive and have a higher absolute value than the additive effects. Therefore, if one White leghorn layer allele appeared by mutation in a Red junglefowl population at any of the two loci, *A* or *B*, it would be maintained at a certain frequency because of balancing selection—superiority of the heterozygote—but it would neither disappear nor reach fixation. This suggests that one mutation could be present at some frequency in the population when the second one appeared.

For analyzing what would happen if eventually the two mutations were present at the same time in the population, we have to consider also the interaction effects. The double homozygote for White leghorn layer allele increases the phenotype with roughly forty grams (four times *aa*, in Table 3 as it can be deduced from **G** = **S**⋅**E**, with the reference of *R* = *G*_1111_), relative to the expected value without epistasis, which is a decrease in roughly 20 grams from the Red junglefowl. In total, this makes the phenotype of the White leghorn layer 20 grams higher than the Red junglefowl. However, for inspecting if this results support the White leghorn layer alleles being likely to reach fixation we also need to consider the phenotypes of the heterozygotes. Interactions involving dominance in locus *B* are all negative, thus favoring the fixation of the White leghorn layer allele, *B*_2_. The role of allele *A*_2_ is not as obvious, since *da* is positive. The genotypic value of “*A*_1_ *A*_2_ *B*_2_ *B*_2_” is roughly 30 grams higher than the Red junglefowl (computed again from Table 3 and **G** = **S**⋅**E**) and ten grams higher than the pure White leghorn layer. The expected, therefore, would be that the two alleles segregate at locus *A*. The standard deviations of the estimates are however rather large and thus do not rule out the possibility of fixation of the White leghorn layer allele at locus *A*.

## Discussion

### The Meaning of the Statistical Estimates

The statistical formulation of NOIA is orthogonal under random deviations from ideal experimental populations and outbreeding pedigrees [16]. Therefore, NOIA can provide meaningful estimates of genetic effects—as allele substitutions made in the population or sample under study—and a proper decomposition of the genetic variance under those circumstances. In this article, we illustrate the practical implications of these achievements for estimation of genetic effects and QTL analysis in two ways. First, we simulated a two-locus genetic system under departure from the HWP affecting one of the loci underlying the trait under study. This scenario can have a biological origin or be due to sampling alone and it is commonly occurring in experimental data both from natural and experimental populations, such as for the QTL pair we have studied (see below). We therefore deemed it relevant to test the performance of NOIA in practice—by assessing how departures from HWP cause other models to deviate from the orthogonal values. Our results show that departures from HWP substantially affect both the genetic effects and the decomposition of variance. The cause for this is that epistasis makes the genetic effects dependent on the genetic background, which is different under different degrees of departures from HWP. NOIA can capture the proper, orthogonal genetic effects, and thus also their orthogonal variances, in the simulated populations whereas the deviances from these values due to using the other—nonorthogonal—models increases with the departures from HWP.

Second, we used experimental data on epistatic QTL from a previously published study [21] to explore how much of a difference it makes to use NOIA instead of previous statistical models, when departures from HWP are not larger than expected by sampling. Even though the population we studied was rather large (approximately 800 individuals), the random deviations from the HWP in this set of available individuals cause considerable differences in the estimates of genetic effects performed with models that would be orthogonal in totally ideal situations, as compared to the estimates obtained using NOIA. These differences become even more noteworthy for the components of variance estimated using the different models. These values influence consequential quantities, like the heritability of one trait, which may be needed for instance for performing artificial selection at the available sample of individuals. Orthogonal models are also important for finding the genetic architecture of traits—albeit this has not been our focus in this communication. In principle, when testing the effect of a particular locus or set of loci in a QTL analysis, the choice of the model of genetic effects to use does not matter. However, it does matter when it comes to compare which of several putative sets of loci is the most likely genetic architecture underlying the trait, i.e., when performing model selection in QTL analysis. This is so because orthogonal models have the convenient property that the estimates and their variances remain the same when considering reduced models, which facilitates model selection strategies [19].

### Translating Estimates To Fit Other Meanings

After model selection and the estimation of genetic effects have been properly carried out using an orthogonal model, the obtained estimates provide the effects of allele substitutions in the sample of individuals used in the study, and the decomposition of variance is also the appropriate one in that particular sample of individuals. The NOIA model provides convenient tools for transforming those estimates into the ones with any other desired meaning, like the orthogonal estimates and the decomposition of variance in a different population [16]. This is useful to compare results from QTL studies performed in different populations, and to use the results obtained with one orthogonal model in one population to study the evolution of the same trait in a different population.

One example of the previous is removing the characteristics of the data that are not supposed to be properties of a target population from the estimates. The departures from HWP of the experimental data we dealt with in this article are in fact supposed to be only due to sampling, instead of being caused by real Hardy-Weinberg disequilibrium in the F_2_ population. If we were interested in the genetic effects or in the decomposition of variance of the ideal F_2_ as a target population—in which the departures from HWP are absent—we could use the transformation tool of NOIA to obtain (from the original estimates with the reference of the mean of the sample population) the ones with the reference of the mean of an ideal F_2_ population. Further, as illustrated in the example with real data, it is possible to transform statistical estimates of genetic effects into functional ones, using a particular reference genotype. Another situation in which these transformations are valuable is, for instance, in a three-locus genetic system with pairwise epistasis. In this case, NOIA would easily permit to consider only the significant genetic effects and to re-compute the genotypic values only from the significant genetic effects (assuming the non-significant third-order interactions to be zero).

### Functional Estimates of Genetic Effects

Statistical models of genetic effects are necessary for QTL analysis and for performing orthogonal decompositions of the genetic variance in populations. Functional models of genetic effects, on the other hand, are convenient—especially in the presence of epistasis—for studying evolutionary properties of the populations such us adaptation in the presence of drift and speciation (see *e.g.* [23],[24]). NOIA is the first model framework that successfully unifies functional and statistical modeling of genetic effects [16]. This enables researchers to feed models of functional genetic effects, so far mainly used in simulation studies (see *e.g.* [2],[24]), with real data obtained using statistical models in QTL mapping experiments. Here, we have actually transformed statistical genetic effects, obtained from real data of an F_2_ experimental population, into functional genetic effects as allele substitutions performed from a reference individual. Concerning these functional estimates of genetic effects, we have shown in the previous section how they can improve the understanding of the genetic system by inspecting a two-locus model obtained from real data. Notice that when changing the reference of the model, the genetic effects can change their magnitudes and even their signs (see Tables 2 and 3). Therefore, for reaching the kind of conclusions we obtain above for the evolution of a population from an ancestral genotype “*A*_1_ *A*_1_ *B*_1_ *B*_1_”, the genetic effects have to be described with a model that uses that particular genotype as reference point. Those are the only ones that are meaningful for analyzing the problem under consideration.

### The HKR with NOIA

The computation of genetic effects using NOIA in the example with real data required the use of the theory developed in this article, the implementation of the model to handle missing data (1). When performing IM for searching for the positions and estimates of genetic effects in QTL mapping experiments, missing data occurs at two levels. First, the genotype of the QTL located in a marker interval is not known and needs to be estimated from the observed flanking marker genotypes. Second, in most experimental datasets there are missing genotypes for many genetic markers that can be imputed from genotypes at closely linked informative markers. Thus, the implementation of HKR with NOIA enables us to perform IM with a regression method and using a model of genetic effects that is orthogonal regardless of how far the available data is from the HWP.

The HKR has been assessed as a good approximation of IM when dense marker maps are available and missing data are few and random [25],[26], but some disadvantages of this method have also been reported. The residual variance of the HKR has been found to be biased, as first pointed out by Xu [27]. Kao [26] further characterized that bias and found it to be noticeable under LD or strong epistasis. Nevertheless, even in those cases, the estimated genetic effects themselves are not biased [26]. Feenstra *et al.* [25] have developed a new method, the estimating equation method, which reduces the reported bias of the HKR and is therefore more suitable in the cases when it has proven to be strongly biased. However, the traditional HKR is still popular and convenient mainly due to its dramatic advantage in computational time [25], and this is why in this study we have chosen this method for implementing NOIA for IM.

### Toward a Completely General Model of Genetic Effects

Models of genetic effects need to be further generalized. Two important cases that need to be accounted for are multiple-alleles and LD, which have been addressed in several recent publications dealing with statistical models of genetic effects. Yang [18] has developed a model to test the importance of LD in QTL data, by designing a component of variance due to LD. This statistical model, like the statistical formulation of NOIA, actually accounts for departures from HWP, although it is restricted to the two-locus case. Wang and Zeng [20] have developed a statistical model with multiple alleles in which they also test the importance of LD, in this case by computing all the covariances between the components of variance, due to LD. It is, however, restricted to HWP. Mao *et al.* [28] have developed a model to account for LD when computing genetic effects in a two-locus model specially designed for single nucleotide polymorphisms. The desired situation, which we are currently aiming toward is to consider all the different departures from ideal situations gathered under the umbrella of a general formal framework of genetic effects.

## Methods

### Genetic Models under Departures from Hardy-Weinberg

We use a simulated numerical example to show how departures from the HWP affect the estimates of genetic effects in several models of genetic effects. We simulate a trait controlled by two biallelic loci, *A* and *B*, generating several populations with the second locus affected by departures from the HWP in several degrees. The genotype-phenotype map corresponds to the phenotype mean of the population and all the genetic effects being equal to one in an ideal F_2_ population (Table 1). We first constructed data for an ideal F_2_ population of 800 individuals in strict HWP and LE. From this population we subsequently removed 24 *A*_2_ *A*_2_ individuals and added eight *A*_1_ *A*_1_ and 16 *A*_1_ *A*_2_ individuals in a balanced way, without affecting the population size, the frequencies at locus *B*, the proportion of *A*_1_ *A*_1_ versus *A*_1_ *A*_2_ individuals or LE. Only deviations from the HWP against the *A*_2_ *A*_2_ homozygote were introduced in the data. We repeated this procedure eight times in total and saved each population data, until only eight *A*_2_ *A*_2_ individuals remained. We measured the departures from HWP in these populations by computing the percentage of reduction of *A*_2_ *A*_2_ individuals relative to *A*_1_ *A*_1_, which of course was zero in the ideal F_2_ population we started from.

We analyzed the simulated data by computing the genetic effects of the system using three models: NOIA, G2A and F_2_. The F_2_ model, described in Text S1, is constructed for F_2_ populations, although it is only orthogonal in ideal F_2_ populations with the genotypic frequencies being exactly ¼, ½, ¼. The NOIA model is as described in Text S1. The G2A model [19] accounts for any gene frequencies of—and it is orthogonal at—populations under exact HWP. Álvarez-Castro and Carlborg [16] obtained it as a particular case of NOIA by constraining (S5), in Text S1, to HWP:
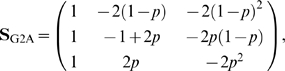
where *p* is the frequency of allele *A*_1_. The genetic effects were computed for each individual genotype using the genetic-effects design matrices and the estimates of genetic effects from each of the three models, which produced different outcomes. The additive, dominance and interaction variances were obtained as the correspondent sums of the variances of each genetic effect (for instance, the sum of the variances of the additive effects of each of the loci gives the additive variance).

### Implementing the Haley-Knott Regression with NOIA

We recall the required theory behind the HKR and NOIA in Text S1. Here we extend the NOIA model to IM with HKR. We do this by implementing the genetic-effects design matrix of the statistical formulation of NOIA, **S** *_S_* (S5), in the HKR method, as we do with the F_2_ model in Text S1. The original genotype frequencies *p*_11_, *p*_12_ and *p*_22_ in the NOIA statistical formulation (S5) are the exact genotype frequencies at the considered loci. In the HKR, the genotype frequencies are not known, but can be estimated as:
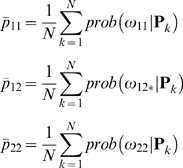
where *N* is the number of individuals in the population under study. We implement this model in the general expression of the HKR (S4), in Text S1, and obtain:
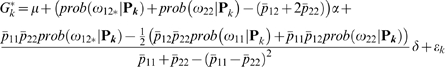
Let **G** ^*^ be the column-vector of observed phenotypes, *G* ^*^ *_k_*, *k* = 1,…,*N*, **ε** the corresponding vector of errors, and **Z**, which is an *N*×3-matrix whose rows are the vectors ω*_k_* (S4). With this notation, the general expression of regression (S4) is:
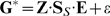
(1)This has a straightforward extension to several loci with LE. The **S** *_S_* matrix and the **E** vector can be extended as in Álvarez-Castro and Carlborg [16]. The **Z** matrix can be extended as the row-wise Kronecker product of the matrices of the single loci, also as in Álvarez-Castro and Carlborg [16], albeit in that article the matrix accounted for only complete marker information, instead of for IM with HKR, or for missing data probabilities. For instance, for a two-locus (*A* and *B*) case, the **Z** *_AB_* matrix is an *N*×9-matrix that is built as:
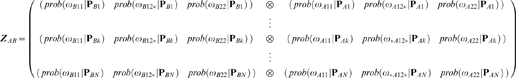



### Experimental Data

Carlborg *et al.* [21] identified 10 genome-wide significant QTL for growth rate in chicken from eight to 46 days of age in an F_2_ intercross of roughly 800 individuals between one Red junglefowl male and three White leghorn females. A simultaneous two-dimensional genome scan was performed to identify pairs of interacting loci regardless of whether their marginal effects were significant or not. We have studied in more detail one of the detected pairs involving QTL on chromosome 2 (486 cM) and 3 (117 cM), hereafter loci *A* and *B* respectively. This pair was selected for a number of reasons. First, these loci interact epistatically, in spite of showing no significant marginal effects in the studied population. Second, since they are located in different chromosomes, there is no physical linkage between them. Third, the genotype frequencies at locus *A* depart significantly from the HWP (*p*<0.05) when considered independently, but the departure is not significant after applying multiple testing correction accounting for the rest of the detected QTL. Thus, locus *A* is an example of the departure of the HWP that is expected in QTL experiments just due to sampling. The level of departure from the HWP for the evaluated pair roughly equals the 30% deviation in Figures 1 and 2.

We have computed the genetic effects of the epistatic pair involving loci *A* and *B*, using several models of genetic effects. First we used the F_∞_ model, which was the one also used by Carlborg *et al.* [21] as it was the model originally implemented in HKR [9],[29]. Second, the F_2_ model, which was designed for F_2_ populations. Third, the G2A model, which can account for departures of the gene frequencies from ½, and finally the statistical formulation of NOIA, which can adapt to the genotype frequencies of the sample used for the estimation of QTL effects. In these analysis we have made use of the theory developed in this article: the implementation of HKR with NOIA. These developments enable us to deal both with missing data and with the estimation of genetic effects of positions inside the marker intervals.

### Transforming Errors of the Estimates in NOIA

Álvarez-Castro and Carlborg [16] have shown how to transform genetic effects obtained using an orthogonal-statistical model in one population, into statistical genetic effects at any other population or into functional genetic effects from any reference individual. In each of these two cases, the transformation is done as in expression (S6), in Text S1, using the **S** matrix—the genetic-effect design matrix—of the orthogonal system, **G** = **S**_1_⋅**E**_1_, and the inverse of the **S** matrix in the new system, **G** = **S**_2_⋅**E**_2_:
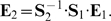
(2)Let
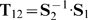
(3)be the transformation matrix. From (2) and (3), the estimates in **E**_1_ can be expressed as functions of the estimates in **E**_2_ as:
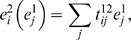
(4)where the letters and their superindexes indicate the vector, or matrix, they are scalars of and the subindexes indicate the position of the scalars inside the vectors or matrices. From (2), the variances of the estimates **E**_2_, can be computed from the ones in **E**_1_ as:
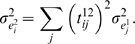
(5)Now for obtaining the vector of variances of the estimates **E**_2_, **V**_2_, from the vector of variances of the estimates **E**_1_, **V**_1_, we just rewrite (3) in algebraic notation as:
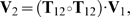
(6)where the open circle stands for the Hadamard product—giving the matrix whose scalars are the product of the scalars at the same position in the original matrices.

## Supporting Information

Text S1 Background information on the HKR and NOIA. Concepts and equations related to the original formulation of the HKR and to the NOIA statistical formulation that will help the reader to deeper understand some details of the methods used in the article.
(0.09 MB DOC)
