# Supplementary material for: How To Perform Meaningful Estimates of Genetic Effects
Source: PLoS Genet. 2008 May 2;4(5):e1000062. doi: 10.1371/journal.pgen.1000062 (PMC2320976; doi:10.1371/journal.pgen.1000062)
Supplement: Text S1 — Background information on the HKR and NOIA. Concepts and equations related to the original formulation of the HKR and to the NOIA statistical formulation that will help the reader to deeper understand some details of the methods used in the article. (0.09 MB DOC) [file pgen.1000062.s001.doc]

BACKGROUND INFORMATION ON THE HKR AND NOIA

**Original formulation of the HKR.**

The original HKR for one locus and two alleles, “1” and “2”, uses the following parameterization of genotypic means, *μ*+*α*, *μ*+*δ*, *μ*−*α*, for the genotypes “11”, “12” and “22” respectively, where *μ* is the mean of the reference population and *α* and *δ*, are the additive and dominance genetic effects . This can be written in terms of index variables as , where:

(S1)

This arbitrary, popular parameterization is called the F_∞_ model [11]. Another—more convenient—way of describing a genetic model consists in expressing the genotypic values of the different genotypes in terms of a genetic-effect design matrix times a vector of genetic effects, $G=S\cdot E$, which for a one-locus two-alleles model becomes:

, (S2)

The F_∞_ model can be expressed in algebraic form (S2) by just using the **S**_F∞_ matrix that can be built from the index variables (S1) as:

. (S3)

To be precise, the **S**_F∞_ matrix in accordance to (S1) differs from (S3) in the signs of the second column. The parameterization (S1) fits to a QTL experimental design in which allele “1” comes from a “high line” (an inbred line that has been selected to increase the phenotypic value of a character under study), and allele “2” comes from a “low line” (selected to decrease the phenotypic value). The additive effect, *α*, stands for the absolute value of the decrease of the genotypic value due to a substitution from allele “1” to allele “2”. On the contrary, in the parameterization of the F_∞_ model we show in (S3), *α* is actually negative in the case mentioned above, thus accounting for the precise effect of the allele substitution from “1” to “2”.

Regardless of the way the F_∞_ model is parameterized, the regression model to analyze the data expresses the genotypes of individual *k* as:

, (S4)

where:

We now use the notation , instead of *G_i_*, to show that the expression describes the real phenotype measured from an individual, individual *k*, with a particular genotype, instead of the genotypic value of a genotype—the expected phenotype of the genotype *i*. This is why the error term, *ε_k_*, is needed for each individual.

To account for the uncertainty in the genotypes in IM, Haley and Knott [9] (see also [29]) modified the vector *ω_k_*, to contain the conditional QTL genotype probabilities given the observed marker data for individual *k*. For the F_∞_ model, and using the notation of Haley and Knott [9], this extends to the original expression of the HKR:

where:

The probabilities:

are conditioned on the genotype of individual *k* at the flanking markers, **P***_k_*, and they are calculated as described by Haley and Knott [9] for inbred lines and Haley *et al.* [29] for outbred lines.

The HKR can also be built with genetic models different from the F_∞_ model. Here we briefly illustrate this by implementing HKR with Conckerham’s F_2_ model of genetic effects. This model is orthogonal for ideal F_2_ populations, with frequencies ¼, ½, ¼ [17], and for this reason it has been implemented in QTL mapping methods (see *e.g.* [19]). Using the formulation in (S4), the HKR can now be implemented to work with the F_2_ model by updating the **S** matrix accordingly. The **S** matrix of the F_2_ model is:

Please note that we are using the signs in the second column that are appropriate for a general interpretation of the additive effects, as discussed above. Using this **S** matrix, (S4) extends to:

In analogy with this, the F_2_ model can be used to parameterize the genetic effects in IM, instead of the F_∞_ model that was originally used by Haley and Knott [9].

**The NOIA statistical formulation.**

The statistical formulation of NOIA is more general than previous statistical models of genetic effects. The **S** matrix of the one-locus statistical formulation of NOIA is:

, (S5)

where *p_ij_*, *i*≤*j*, are the frequencies of the three genotypes, “11”, “12” and “22” [16].

This is a general orthogonal formulation regarding the genotype frequencies of the population considered and it comprises other models of genetic effects, like the F_∞_ and the F_2_ models, as particular cases. This is possible because expression (S5) accounts for the frequencies of the population under study, being for instance ¼, ½, ¼ for the F_2_ model, as mentioned above. The algebraic notation allows to make a straightforward extension of the model to several loci under LE by just computing the Kronecker product of the **S** matrices of each locus and extending the vectors of genotypic values and genetic effects accordingly [16].

The generality of the model, together with the algebraic notation, makes it possible to develop a powerful change-of-reference tool to translate the genetic effects measured in one population to how they would look like if measured in another population with different genotype frequencies. This allows us, in particular, to estimate genetic effects for one trait using different orthogonal matrices for different particular experimental populations (accounting for even the specific sampling errors in each of them), and to then compare the results of all the different experiments by translating all the estimates of genetic effects into the same population, say an ideal F_2_ population. This translation of genetic effects from the population under study to the F_2_ population can be done as,

, (S6)

where the subindex *μ* indicates the population under study [16].
